# Local Delivery of Nimodipine by Prolonged-Release Microparticles—Feasibility, Effectiveness and Dose-Finding in Experimental Subarachnoid Hemorrhage

**DOI:** 10.1371/journal.pone.0042597

**Published:** 2012-09-25

**Authors:** Daniel Hänggi, Jason Perrin, Sven Eicker, Kerim Beseoglu, Nima Etminan, Marcel Alexander Kamp, Hi-Jae Heiroth, Nadia Bege, Stephan Macht, Katrin Frauenknecht, Clemens Sommer, Thomas Kissel, Hans-Jakob Steiger

**Affiliations:** 1 Department of Neurosurgery, Heinrich-Heine-University, Düsseldorf, Germany; 2 Institute of Neuroradiology, Heinrich-Heine-University, Düsseldorf, Germany; 3 Department of Pharmaceutics and Biopharmacy, Philipps-University, Marburg, Germany; 4 Department of Neuropathology, University Medical Center of the Johannes-Gutenberg-University, Mainz, Germany; University of Münster, Germany

## Abstract

**Background and Purpose:**

To investigate the effect of locally applied nimodipine prolonged-release microparticles on angiographic vasospasm and secondary brain injury after experimental subarachnoid hemorrhage (SAH).

**Methods:**

70 male Wistar rats were categorized into three groups: 1) sham operated animals (control), 2) animals with SAH only (control) and the 3) treatment group. SAH was induced using the double hemorrhage model. The treatment group received different concentrations (20%, 30% or 40%) of nimodipine microparticles. Angiographic vasospasm was assessed 5 days later using digital subtraction angiography (DSA). Histological analysis of frozen sections was performed using H&E-staining as well as Iba1 and MAP2 immunohistochemistry.

**Results:**

DSA images were sufficient for assessment in 42 animals. Severe angiographic vasospasm was present in group 2 (SAH only), as compared to the sham operated group (p<0.001). Only animals within group 3 and the highest nimodipine microparticles concentration (40%) as well as group 1 (sham) demonstrated the largest intracranial artery diameters. Variation in vessel calibers, however, did not result in differences in Iba-1 or MAP2 expression, i.e. in histological findings for secondary brain injury.

**Conclusions:**

Local delivery of high-dose nimodipine prolonged-release microparticles at high concentration resulted in significant reduction in angiographic vasospasm after experimental SAH and with no histological signs for matrix toxicity.

## Introduction

Angiographic vasospasm and delayed cerebral ischemia (DCI) are major contributors for secondary morbidity and mortality following aneurismal subarachnoid hemorrhage (SAH). [1], [2] Despite the current pharmaceutical treatment strategies permanent disability and death following DCI are still estimated 10–20%. [3] Therefore, more effective treatment regimens are still required.

In addition to the only proven therapy, i.e. orally administered nimodipine, surgically implanted nicardipine prolonged-release pellets have also been suggested to be effective in experimental as well as clinical studies to reduce DCI, cerebral infarction and to improve clinical outcome in patients suffering from aneurismal SAH. [4], [5] However, a major limitation of the pellets is the necessity for microsurgical application via a craniotomy, thus restricting this beneficial treatment to surgically-treated patients. [5] Additionally, pellets are prepared with dimethylchloride, a neurotoxin that may not be optimal for human use. In view of this limitation, a new and liquid nimodipine microparticles loaded Tisseel® matrix was developed and analyzed in vitro. *A* possible administration in surgically as well as in endovascularly treated patients, e.g. via an external ventricular drain was considered as the major advantage, as compared to aforementioned nicardipine pellets.

The purpose of this first experimental study on the nimodipine prolonged-release microparticle matrix for experimental SAH was to analyze its safety, feasibility and effectiveness for reduction of angiographic vasospasm and incidence of secondary brain injury.

## Materials and Methods

### Experimental Design

All animal experiments were performed in strict accordance with the recommendations in the Guide for the Care and Use of Laboratory Animals of the National Institutes of Health. The experimental study was reviewed and approved by the local Committee for Animal Experimentation, Düsseldorf, Germany (approval no. 8.87-50.10.34.08.246).

A total of 70 male Wistar rats weighing 250–350 g were used for this study. The 70 animals were housed under a light/dark cycle with free access to food and water and randomized (manual randomization technique) into three groups: 1) Sham operated group (baseline); n = 20, 2) SAH group; n = 20 and 3) SAH plus nimodipine microparticles group; n = 30. In detail, the nimodipine microparticles were applied using three different concentrations, 20% concentration group; n = 10, 30% concentration group; n = 10 and 40% concentration group; n = 10 (see below). The experimental SAH and the surgical implantation procedure were performed on days 1 and 2. Neurological condition was assessed daily according to a modified Bederson grading scale circling to one side. [6] Cerebral catheter angiography was performed on day 5, followed by sacrifice of the animals and harvesting of brain specimen.

The investigators performing surgeries and analysis of behavioral, radiographic and histological endpoints were blinded to treatment group allocation.

### Rat double SAH model and surgical implantation technique

All invasive procedures were performed under general anaesthesia with intraperitoneal application of xylazine hydrochloride (10 mg/kg body weight, Vetoquinol, Germany) and ketamine, (100 mg/kg body weight, Pfizer, Germany) allowing spontaneous breathing. The body temperature was kept at 37°C. Surgical procedures were performed under sterile conditions. Cerebral vasospasm was induced by double blood injection into the cisterna magna. [7], [8] After positioning and fixation of the animal, the atlanto-occipital membrane was surgically exposed. The cisterna magna was punctured under microsurgical view using a 27 G needle, after which 0.2 ml cerebrospinal fluid was aspirated with subsequent injection of 0.2 ml autologous blood. The animals were then positioned head down for ten minutes to avoid leakage of injected blood and the surgical wound was closed. The same procedure was repeated on day 2. In animals belonging to the sham-operated group, the atlanto-occipital membrane was solely exposed, the animal was positioned head down for ten minutes and the surgical wound was closed. This procedure was repeated on day 2. The intracisternal application of the nimodipine microparticle loaded Tisseel® matrix was performed after finishing the head down positioning sequence, following the second blood injection.

### Nimodipine microparticle loaded matrix

Poly-D,L-lactide-*co*-glycolide (PLGA, Resomers® RG502H) were obtained from Boehringer Ingelheim (Ingelheim, Germany). Nimodipine was purchased from Sigma-Aldrich (Steinheim, Germany). The fibrin sealant Tisseel® (Duo S 0.5 mL Immuno) was acquired from Baxter (Unterschleissheim, Germany). All other chemicals and solvents used were of analytical grade.

### Preparation of *in situ* forming devices

**Figure 1:**
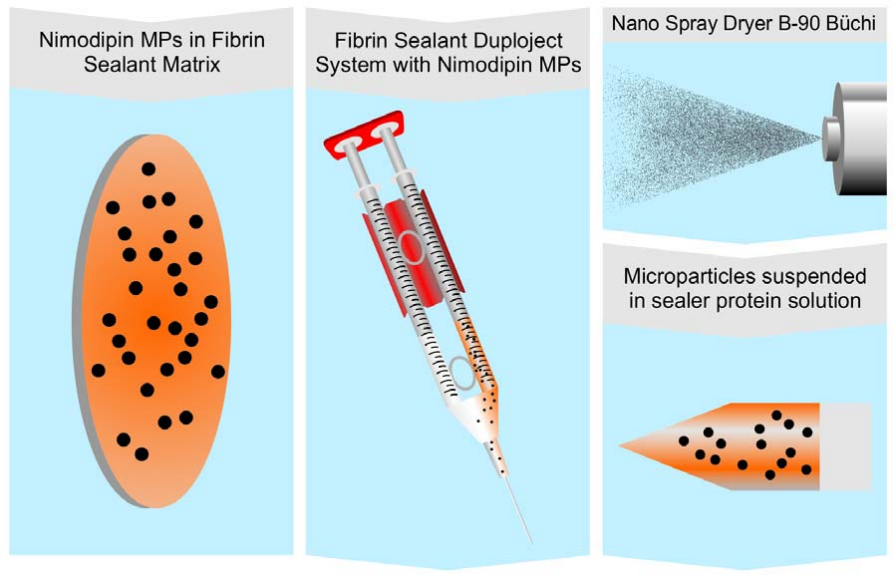
Preparation of *in situ* forming nimodipine depot system.

First, all microparticle formulations were prepared using the spray drying method and the Nano Spray Dryer B-90 (Büchi, Flawil, Switzerland). [9], [10] Different loadings of nimodipine PLGA particles were manufactured: 300 mg solid containing 20%, 30% or 40% (w/w) nimodipine and PLGA were dissolved in 50 mL dichloromethane. The resulting spraying solutions were cooled and spray-dried using the following conditions: 120 L/min drying gas flow rate, 45°C inlet temperature, 50% relative spray rate, 46 mbar pressure and 7.0 um spray mesh. During spraying additional 10 mL dichloromethane were added to restock evaporated solvent. Different concentrations of Nimodipine loaded PLGA particles were then prepared (20%, 30%, 40% w/w). Second, the formulations were suspended in Tisseel® fibrin sealant to create the liquid *in situ* forming device. Therefore, 24 mg particles were mixed with 0.5 mL of sealer protein solution via vortexing. To remove air bubbles, the suspension was sonicated. After vortexing for a second time, the suspension was drawn up into the empty sealer protein syringe and assembled in to the duploject system of Tisseel®, again. The preparation of the microparticle loaded Tisseel® matrix was done immediately before application in rats (*Figure 1*).

### Effectiveness of *in situ* forming devices

The preparation of in situ forming devices have demonstrated incorporation of nimodipine loaded biodegradable polymer microparticles into Tisseel® fibrin sealant. Smooth and round-shaped nimodipine PLGA microparticles with high encapsulation efficiency were suspended in fibrin sealant. This system had built an in situ forming device were the particles were homogeneously arranged and immobilized in this second matrix system. Thermal analysis revealed solubility of nimodipine in the PLGA matrix.

### Angiographic assessment

The angiographic studies were also performed under intraperitoneal anaesthesia as aforementioned. After positioning the animal supine, a cervical midline incision was made and the common carotid artery was exposed. This artery was tapped using a small needle attached to a microcatheter (27 G needle and Prowler 14 microcatheter (Cordis)), and the angiography was performed under manual pressure controlled injection of 0.1 ml contrast agent (Ultravist 300, Schering, Germany) in our clinical angiography suite (Integris Allura, Philips, Netherlands). Angiography was repeated up to four times to achieve images free of respiration artefacts. Artery segments in the DICOM data sets were then measured using computerized quantification of grey values, a method that was shown to correlate exactly with the cross-sectional area of the vessel itself in vitro and in vivo models. [11] The mean perimeters (cross-sectional area) of defined segments of arteries were expressed as percentage of lumen patency in comparison to the individual stapedial artery. The animals were sacrificed after the angiography by intraperitoneal injection of sodium pentobarbital (200 mg/kg body weight, Sanofi-Aventis, Germany).

### Immunohistochemistry of cerebral specimen

Morphological analysis was performed on brains from 22 rats (sham n = 3; SAH vehicle n = 4; SAH 20% n = 5; SAH 30% n = 6; SAH 40% n = 4). Brain specimen were serially cut on a cryostat at −20°C into 20 µm thick horizontal sections (stereotaxic coordinates interaural 4.68; Bregma −5.32) and stained with hematoxilin and eosin according to standard protocols. [12] One section per brain was processesed for Iba-1 and MAP2 immunohistochemistry as follows: After fixation with 4% paraformaldeyhde for 1 h (Iba1) or 10 min (MAP2), the sections were washed once for 10 min with PBST. To block endogenous peroxidase, sections were then incubated for 30 min with normal goat serum followed by incubation with an anti-Iba-1 serum (polyclonal, rabbit, 1∶1000, No. 019-19741, Wako) or with an anti-MAP2-antibody (polyclonal, rabbit, 1∶200; No. 188 002, Synaptic Systems) over night. After washing with PBST, sections were incubated with biotinylated anti-rabbit antiobody. Immunoreactivity was visualized by the avidin-biotin complex method. Sections were developed in daiminobenzidine (Sigma, St. Louis, USA). Negative controls were obtained by omitting the primary antibody.

To check for subtle ischemic damage, MAP2-immunostained sections were semiquantitatively analyzed. [13] Briefly, sections were scanned under equal lighting conditions at a magnification of ×1.6 with a Leica microscope (Leica, Germany), digitized with the MCID image analysis system (Imaging Research Inc, St. Catharines, Ontario, Canada) and transferred to a computer screen. Analysis of immunoreactivity (IR) for MAP2 was performed in the posterior cortex. The region of interest (ROI) was identified and marked on the monitor, the gray values were automatically assessed by the imaging software (MCID image analysis system; Imaging Research Inc., St. Catharines, Ontario, Canada). The optical density (OD) of the corpus callosum control was used as reference value for background staining (OD_USP_) and subtracted from total OD in the respective ROI (OD_TOT_), resulting in specific OD (OD_SP_) as recently described [26]. OD values were expressed as mean IR ± SD. Analyses were performed by an independent researcher (K.F) blinded to the experimental groups. Values are means of IR ± SD presented as % of sham rats.

### Statistical analysis

To determine statistical significance, MAP2-immunoreactivuity was compared between the different treatment groups using Kruskal-Wallis test. Statistical analysis was done using SPSS for Windows version 15.0.1 (©SPSS Inc. 2006). For statistical analysis of angiographical results tests were chosen according to scale level of variables with t-test to determine difference of the mean in metric or continuous variables and nonparametric tests for ordinal variables. Statistical significance was assumed at p<0.05.

## Results

### Clinical evaluation and angiographic analysis

18 animals died immediately after the double hemorrhage injection or during the course of the experiment (sham-group n = 1, SAH-group n = 8, treatment-group n = 9). The clinical evaluation revealed no delayed neurological deficit over the observation period of 5 days. All animals were killed on day five after the index bleeding.

**Figure 2:**
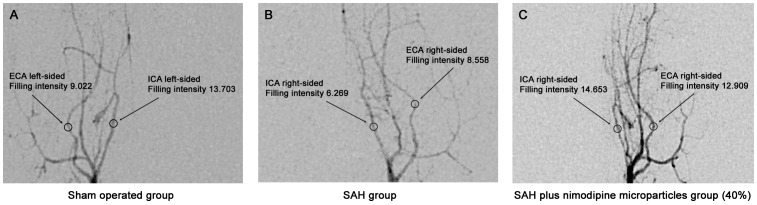
Representative digital subtraction angiographies of A: Scham-operated group, B: SAH group and C: Treatment-group (40%) demonstrating the internal carotid artery (ICA) to external carotid artery (ECA).

Overall, 93 angiographic examinations were performed in 42 animals according to the 3 experimental groups (sham-group n = 14, SAH-group n = 13, treatment-group n = 15). Angiographic evaluation was not possible in 10 animals due to technical problems or mortality due to the angiography itself. Out of these the most prominent angiogram of each animal was chosen for further evaluation by an observer blinded to the treatment groups *(* *Figure 2* *)*.

**Figure 3:**
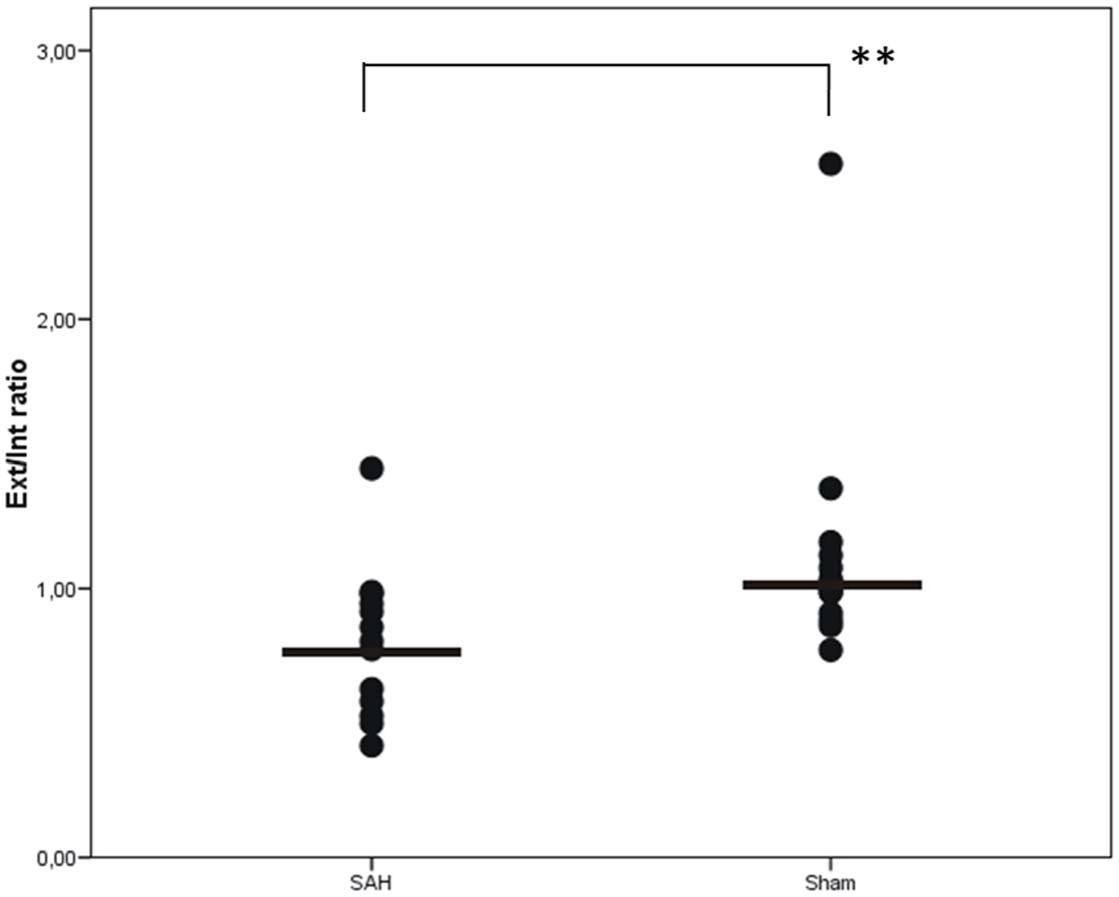
Angiographic comparison of the sham-operated and the subarachnoid hemorrhage (SAH)-only group, the angiographic comparison of the sham-operated group and the subarachnoid hemorrhage (SAH)-only group revealed a significant difference of arterial filling (p = 0.004, Kruskal-Walis-test).

Statistically significant reduction of arterial diameter was induced with the double hemorrhage model (SAH only Group, p = 0.004, Kruskal-Walis-test; *Figure 3*).

**Figure 4:**
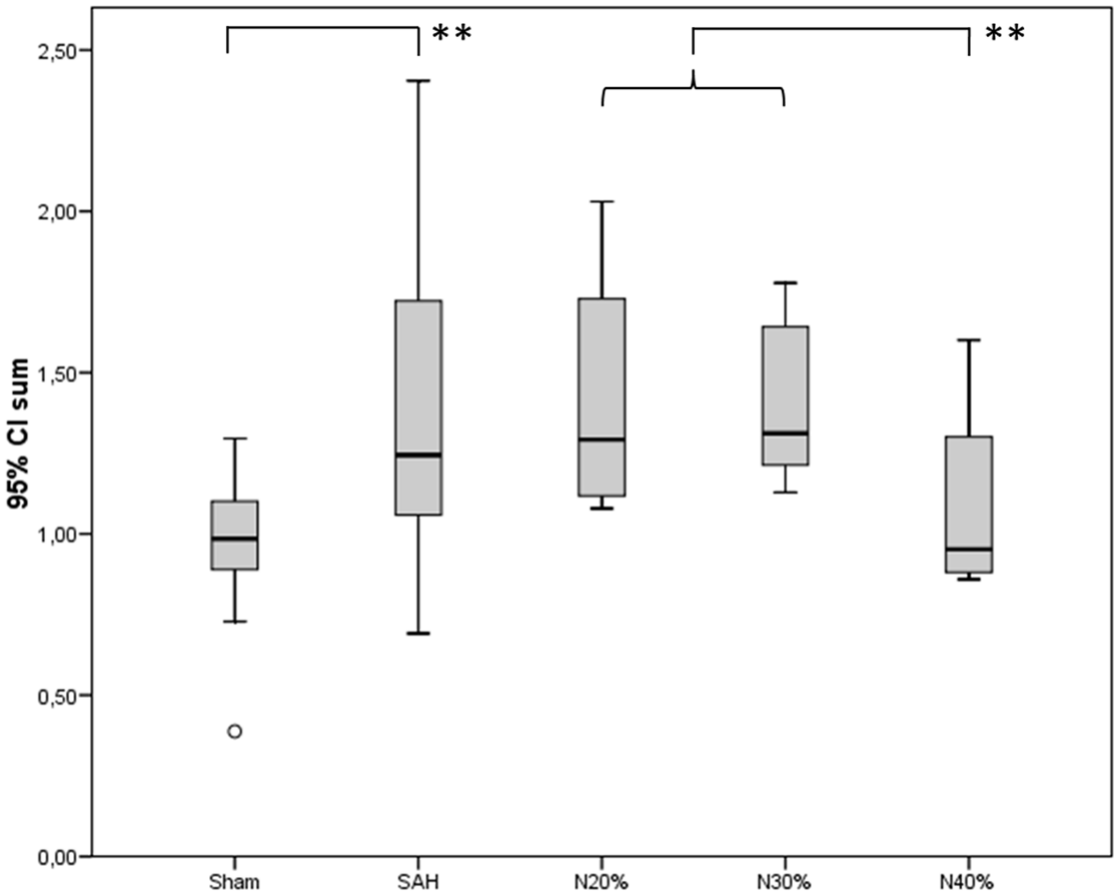
Graph showing mean value of the total brain cross-sectional vessel area (grey bars) with confidence interval (black lines) in all groups, the treatment group receiving 40% of nimodipine microparticles demonstrated the highest filling intensity, with a statistically significant difference to the treatment group receiving 20% and 30% of nimodipine microparticles (p = 0.014, Mann-Whitney test).

There was a significant difference in relative intracranial filling intensity among the groups (p = 0.005, Kruskal-Wallis test). The treatment group receiving 40% of nimodipine microparticles demonstrated the highest filling intensity, with a statistically significant difference to the treatment group receiving 20% and 30% of nimodipine microparticles (p = 0.014, Mann-Whitney test). There was no difference in filling intensity between the groups treated with 20% and 30% of nimodipine microparticles, as compared to the sham-operated and SAH only group (*Figure 4*).

### Morphological examination

**Figure 5:**
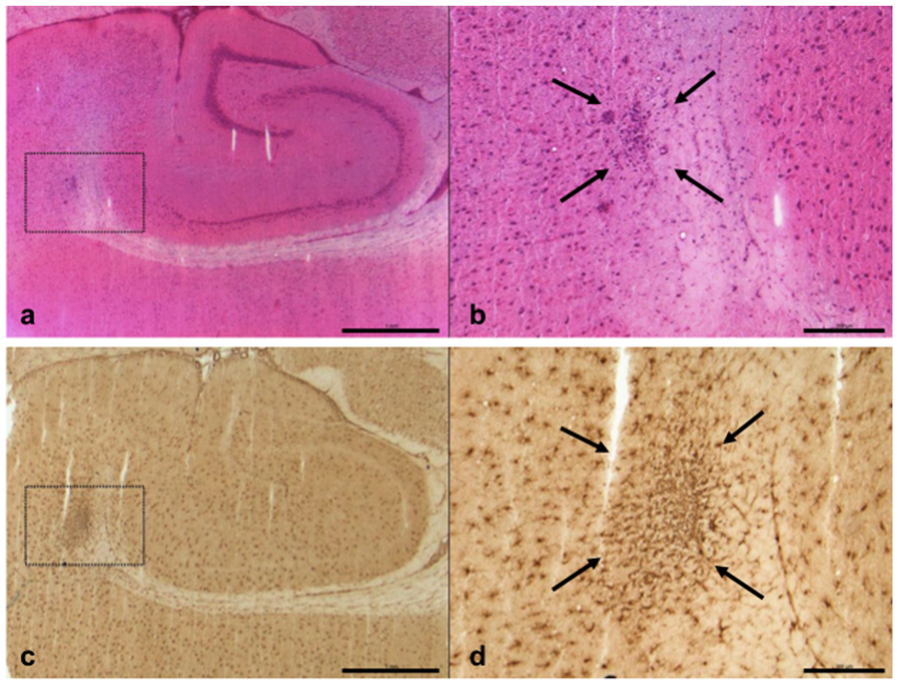
Cortical microinfarct in a control animal. In the H&E stained section, a small infarct in the deeper cortical layers is visible (a). At higher magnification, a mononuclear infiltrate can be distinguished (b, arrows; inset from a), indicating ongoing resorption. Iba1 immunohistochemistry (c; d inset from c) reveals activated microglia in and around the lesion (d, arrows). Scale bar corresponds to 1000 µm (a,c) and 200 µm (b,d), respectively.

**Figure 6:**
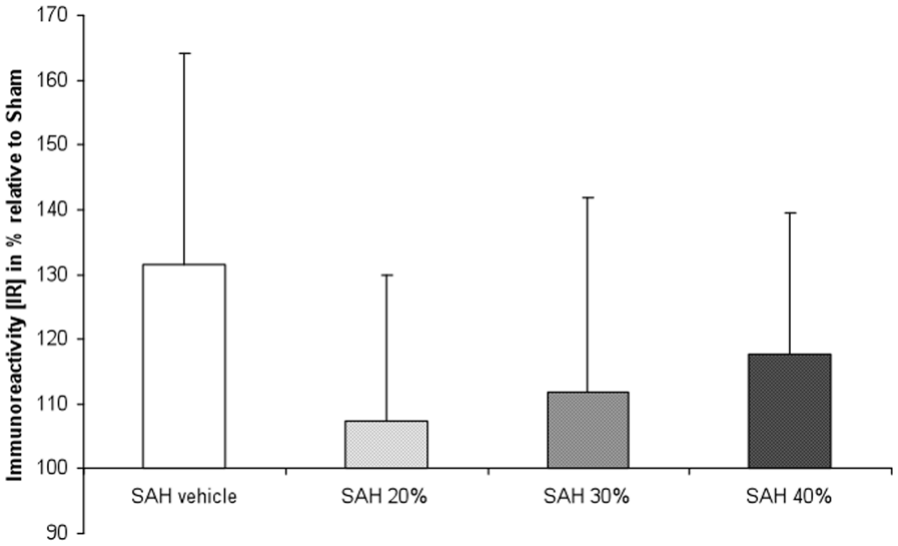
Semiquantitative analysis of MAP2 immunohistochemistry as a marker for subtle ischemic damage reveals no diffrences between the various treatment groups (Kruskal-Wallis test; p<0.05 was considered significant. Values are means of optical density ± SD presented as % of sham rats).

After sacrifice and preparation of the animals, the matrix was localized within the region of the cisterna magna. Neuropathologcial evaluation of the H&E stained sections revealed subarachnoidal blood around the hippocampi in one rat from the SAH 20% and in one rat of the SAH 30% group, while no obvious SAH was visible in the other animal. One rat of the untreated SAH group showed two cortical lesions with dense mononuclear infiltrates (Figure 5). Inflammatory infiltrates were not detectabel in the subarchnoid space. Analysis of the Iba-1 immunohistochemistry confirmed the two lesions seen in H&E plus an additional focus with accumulation of Iba-1 positive microglia, thus resembling small cortical infarcts. Iba-1 positive microglia was evenly distributed with no further lesions in the cerebral sections of all other rats from the various experimental groups. Iba-1 positive microglia was evenly distributed and no lesions were detectable. Semiquantitative analysis of MAP2 protein expression as a sensitive marker for ischemic damage revealed not significantly different levels of optical density among the various experimental groups (Sham, 132±33%; SAH 20%, 107±23%; SAH 30%, 112±30%; SAH 40%, 118±22%) compared to sham rats (100±23%) (Kruskal-Wallis test, p = 0.198) (Figure 6). Furthermore there was no inflammatory response within the subarachnoid space neither due to preparation or the nimodipine matrix system.

## Discussion

The purpose of the present experimental study was to analyze the safety, feasibility and effectiveness of a newly developed nimodipine prolonged-release microparticles matrix after experimental SAH in rats. Pharmacological integrity of our nimodipine prolonged-release microparticles matrix has been previously demonstrated in vitro. Our experimental treatment, i.e. the local delivery of the high-dose nimodipine prolonged-release microparticles, resulted in significant reduction in angiographic vasospasm after experimental SAH. Further, the histological analysis revealed no signs of toxicity of the experimental treatment. The possible administration in surgically as well as in endovascularly treated patients via an external ventricular drain was considered as the major advantage, as compared to aforementioned nicardipine pellets.

Several studies previously investigated experimental SAH in the rat. [14] For the present experimental study, we chose the model of direct injection of autologous blood into the cisterna magna because of the possibility of combined SAH induction and subsequent nimodipine microparticles application during one surgery. Moreover, we decided to use the “double hemorrhage” model due to its increased vasospastic impact. [14] Using this model, experimental vasospasm can be reliably induced and it has been described that the degree of vasospasm in this model relates to the amount of injected blood. [14], [15], [16] For the “double hemorrhage” model, peak angiographic vasospasm and perfusion deficits in this model have been demonstrated to occur on day 5 after induction of SAH. [17] [18] Furthermore the mortality rate in the present study was 26%, which is in line with previous published studies using this hemorrhage model. [17], [19], [20]

The effectiveness of intrathecally-administered nimodipine has been reported in experimental models of SAH in canines, rabbits, monkeys, dogs and rats. One study demonstrated in a group of 8 dogs that a single shot intraventricular application of 100 µg of nimodipine could significantly reduce angiographic vasospasm in the basilar artery. [21] In similar experiments, the results illustrated that single-shot, intrathecal application of nimodipine promptly reversed vasospasm in the canine SAH model. [22], [23] Conversely, one experiment concluded that intracisternal nimodipine (1 ml/0.2 mg) delivered through an Ommaya reservoir in the monkey SAH model did not result in any significant relaxation of cerebral arteries. [24] Only one experimental trial investigated a prophylactic continuous intrathecal application of nimodipine via miniosmotic pumps in the rabbit SAH model. [25] This study demonstrated that prophylactic continuous intrathecal administration of either glyceroltrinitrate or nimodipine (dose of 0.2 mg/ml with a flow rate of 10 µl/h) prevents SAH induced vasospasm. A dose-related efficacy of a continuous intracisternal nimodipine treatment using miniosmotic pumps on cerebral vasospasm in the rat double SAH model has been previously described. [18] Based on our angiographic analysis, a strong dose-dependant effect of our experimental treatment on cerebral vessel diameter was detected. For the groups treated with 20% (0.96 mg) and 30% (1.44 mg) of nimodipine prolonged-release microparticles, the vessel size as documented by DSA did not show a significant difference, as compared to animals belonging to the SAH group. Interestingly, for animals treated with 40% (1.92 mg) of nimodipine prolonged-release microparticles, vessel size analysis revealed a complete reversal of the induced vasospasm. This is in line with previous published experimental and clinical trials supporting the potential efficacy of continuous, intrathecal application of nimodipine to prevent cerebral vasospasm after SAH. [21], [22], [23], [26], [27]

One limitation in the majority of SAH animal models is the lack of morphological ischemic damage, despite effective induction of angiographic vasospasm due to abundant collateral blood flow. [8] However, in our present study, one rat with untreated SAH had developed small cortical infarcts, which was verified after Iba-1 immunohistochemistry. [28] However, microglia were evenly distributed in all other brains investigated and no differences between the various experimental groups were detectable. Additionally, there were no indirect signs of tissue damage due to nimodipine prolonged-release microparticles treatment when using MAP2 immunhistochemistry (Figure 6). Further, angiographic evaluation of small vessels remains difficult, despite various propagated techniques. [15], [29], [30], [31] In this study, we used our validated software-based, measurement tool for analysis of small cerebral vessels and were able to reliably document the aforementioned angiographic diameter difference, in relation to the dose of our study drug. [11] Additionally, the sample size in our study might have been too small to illustrate significant differences in angiographic vasospasm between the different doses of our experimental treatment. Lastly, a vehicle group was not used in the present experiment and further experimental trials will require pharmacokinetic analysis of CSF and plasma concentration for nimodipine.

In summary, the local delivery of high dose nimodipine prolonged-release microparticles after successful induction of experimental SAH resulted in significant reduction of angiographic vasospasm. Based on these results, a dose-dependant effect of our experimental drug must be assumed. Despite the absence of morphological evidence against ischemic events in this model, our data affirms the beneficial effect of locally applied nimodipine on cerebral vasospasm. Moreover, the continuous and prolonged release of nimodipine using microparticles might be a very promising treatment to overcome secondary cerebral injury in patients suffering from SAH.

## Conclusion

Local delivery of high-dose nimodipine prolonged-release microparticles resulted in significant reduction in angiographic vasospasm after experimental SAH. Additionally, the histological analysis revealed no signs of toxicity of the matrix system. This data underlines the necessity for the introduction of clinical trials on nimodipine prolonged-release microparticles in patients with endovascular or surgical treatment for aneurysmal SAH.

## References

[1] Lovelock CE, Rinkel GJ, Rothwell PM (2010) Time trends in outcome of subarachnoid hemorrhage: Population-based study and systematic review. Neurology 74: 1494–1501. 20375310 10.1212/WNL.0b013e3181dd42b3 PMC2875923

[2] Suarez JI, Tarr RW, Selman WR (2006) Aneurysmal subarachnoid hemorrhage. N Engl J Med 354: 387–396. 16436770 10.1056/NEJMra052732

[3] Hop JW, Rinkel GJ, Algra A, van Gijn J (1997) Case-fatality rates and functional outcome after subarachnoid hemorrhage: a systematic review. Stroke 28: 660–664. 9056628 10.1161/01.str.28.3.660

[4] Barth M, Capelle HH, Weidauer S, Weiss C, Munch E, et al (2007) Effect of nicardipine prolonged-release implants on cerebral vasospasm and clinical outcome after severe aneurysmal subarachnoid hemorrhage: a prospective, randomized, double-blind phase IIa study. Stroke 38: 330–336. 17185636 10.1161/01.STR.0000254601.74596.0f

[5] Kasuya H, Onda H, Sasahara A, Takeshita M, Hori T (2005) Application of nicardipine prolonged-release implants: analysis of 97 consecutive patients with acute subarachnoid hemorrhage. Neurosurgery 56: 895–902; discussion 895–902. 15854236

[6] Bederson JB, Levy AL, Ding WH, Kahn R, DiPerna CA, et al (1998) Acute vasoconstriction after subarachnoid hemorrhage. Neurosurgery 42: 352–360; discussion 360–352. 9482187 10.1097/00006123-199802000-00091

[7] Grasso G (2004) An overview of new pharmacological treatments for cerebrovascular dysfunction after experimental subarachnoid hemorrhage. Brain Res Brain Res Rev 44: 49–63. 14739002 10.1016/j.brainresrev.2003.10.003

[8] Megyesi JF, Vollrath B, Cook DA, Findlay JM (2000) In vivo animal models of cerebral vasospasm: a review. Neurosurgery 46: 448–460; discussion 460–441. 10690735

[9] Lee SH, Heng D, Ng WK, Chan HK, Tan RB (2011) Nano spray drying: a novel method for preparing protein nanoparticles for protein therapy. Int J Pharm 403: 192–200. 20951781 10.1016/j.ijpharm.2010.10.012

[10] Li X, Anton N, Arpagaus C, Belleteix F, Vandamme TF (2010) Nanoparticles by spray drying using innovative new technology: the Buchi nano spray dryer B-90. J Control Release 147: 304–310. 20659510 10.1016/j.jconrel.2010.07.113

[11] Turowski B, Hanggi D, Beck A, Aurich V, Steiger HJ, et al (2007) New angiographic measurement tool for analysis of small cerebral vessels: application to a subarachnoid haemorrhage model in the rat. Neuroradiology 49: 129–137. 17111162 10.1007/s00234-006-0168-y

[12] Paxinos G, Watson C (2007) The rat brain stereotaxic coordinates. Elsevier, Academic Press.

[13] Tanay E, Mundel P, Sommer C (2006) Short-term ischemia usually used for ischemic preconditioning causes loss of dendritic integrity after long-term survival in the gerbil hippocampus. Brain Res 1112: 222–226. 16876767 10.1016/j.brainres.2006.07.004

[14] Prunell GF, Mathiesen T, Svendgaard NA (2004) Experimental subarachnoid hemorrhage: cerebral blood flow and brain metabolism during the acute phase in three different models in the rat. Neurosurgery 54: 426–436; discussion 436–427. 14744290 10.1227/01.neu.0000103670.09687.7a

[15] Delgado TJ, Brismar J, Svendgaard NA (1985) Subarachnoid haemorrhage in the rat: angiography and fluorescence microscopy of the major cerebral arteries. Stroke 16: 595–602. 4024174 10.1161/01.str.16.4.595

[16] Verlooy J, Van Reempts J, Haseldonckx M, Borgers M, Selosse P (1992) The course of vasospasm following subarachnoid haemorrhage in rats. A vertebrobasilar angiographic study. Acta Neurochir (Wien) 117: 48–52. 1514428 10.1007/BF01400635

[17] Vatter H, Weidauer S, Konczalla J, Dettmann E, Zimmermann M, et al (2006) Time course in the development of cerebral vasospasm after experimental subarachnoid hemorrhage: clinical and neuroradiological assessment of the rat double hemorrhage model. Neurosurgery 58: 1190–1197; discussion 1190–1197. 16723899 10.1227/01.NEU.0000199346.74649.66

[18] Hanggi D, Eicker S, Beseoglu K, Rapp M, Perrin J, et al (2009) Dose-related efficacy of a continuous intracisternal nimodipine treatment on cerebral vasospasm in the rat double subarachnoid hemorrhage model. Neurosurgery 64: 1155–1159; discussion 1159–1161. 19487896 10.1227/01.NEU.0000340685.06407.FD

[19] Aladag MA, Turkoz Y, Sahna E, Parlakpinar H, Gul M (2003) The attenuation of vasospasm by using a sod mimetic after experimental subarachnoidal haemorrhage in rats. Acta Neurochir (Wien) 145: 673–677. 14520547 10.1007/s00701-003-0052-z

[20] Satoh M, Parent AD, Zhang JH (2002) Inhibitory effect with antisense mitogen-activated protein kinase oligodeoxynucleotide against cerebral vasospasm in rats. Stroke 33: 775–781. 11872903 10.1161/hs0302.103734

[21] Voldby B, Petersen OF, Buhl M, Jakobsen P, Ostergaard R (1984) Reversal of cerebral arterial spasm by intrathecal administration of a calcium antagonist (nimodipine). Acta Neurochir (Wien) 70: 243–254. 6546832 10.1007/BF01406653

[22] Gioia AE, White RP, Bakhtian B, Robertson JT (1985) Evaluation of the efficacy of intrathecal nimodipine in canine models of chronic cerebral vasospasm. J Neurosurg 62: 721–728. 3838768 10.3171/jns.1985.62.5.0721

[23] Zabramski JM, Spetzler RF, Bonstelle C (1986) Chronic cerebral vasospasm: effect of volume and timing of hemorrhage in a canine model. Neurosurgery 18: 1–6. 3945370 10.1227/00006123-198601000-00001

[24] Lewis PJ, Weir BK, Nosko MG, Tanabe T, Grace MG (1988) Intrathecal nimodipine therapy in a primate model of chronic cerebral vasospasm. Neurosurgery 22: 492–500. 3362315 10.1227/00006123-198803000-00007

[25] Marbacher S, Neuschmelting V, Graupner T, Jakob SM, Fandino J (2008) Prevention of delayed cerebral vasospasm by continuous intrathecal infusion of glyceroltrinitrate and nimodipine in the rabbit model in vivo. Intensive Care Med 34: 932–938. 18214428 10.1007/s00134-008-0995-x

[26] Auer LM, Ito Z, Suzuki A, Ohta H (1982) Prevention of symptomatic vasospasm by topically applied nimodipine. Acta Neurochir (Wien) 63: 297–302. 7102422 10.1007/BF01728885

[27] Hanggi D, Beseoglu K, Turowski B, Steiger HJ (2008) Feasibility and safety of intrathecal nimodipine on posthaemorrhagic cerebral vasospasm refractory to medical and endovascular therapy. Clin Neurol Neurosurg 110: 784–790. 18554777 10.1016/j.clineuro.2008.05.001

[28] Frauenknecht K, Bargiotas P, Bauer H, von Landenberg P, Schwaninger M, et al (2010) Neuroprotective effect of Fn14 deficiency is associated with induction of the granulocyte-colony stimulating factor (G-CSF) pathway in experimental stroke and enhanced by a pathogenic human antiphospholipid antibody. J Neuroimmunol 227: 1–9. 20557950 10.1016/j.jneuroim.2010.05.043

[29] Boullin DJ, Aitken V, du Boulay GH, Tagari P (1981) The calibre of cerebral arteries of the rat studied by carotid angiography: a model system for studying the aetiology of human cerebral arterial constriction after aneurysmal rupture. Neuroradiology 21: 245–252. 7266859 10.1007/BF02100154

[30] Longo M, Blandino A, Ascenti G, Ricciardi GK, Granata F, et al (2002) Cerebral angiography in the rat with mammographic equipment: a simple, cost-effective method for assessing vasospasm in experimental subarachnoid haemorrhage. Neuroradiology 44: 689–694. 12185547 10.1007/s00234-002-0781-3

[31] Ono S, Date I, Nakajima M, Onoda K, Ogihara K, et al (1997) Three-dimensional analysis of vasospastic major cerebral arteries in rats with the corrosion cast technique. Stroke 28: 1631–1637; discussion 1638. 9259761 10.1161/01.str.28.8.1631

